# Hepatic flares, their immune signatures, and ALT variability after nucleos(t)ide analogue cessation in HBeAg-negative hepatitis B

**DOI:** 10.1016/j.jhepr.2026.101875

**Published:** 2026-04-29

**Authors:** Marte Holmberg, Annika Niehrs, Olav Dalgard, Nega Berhe, Hailemichael Desalegn, Soo Aleman, Nina Weis, Tore Stenstad, Lars Heggelund, Ellen Samuelsen, Lars Normann Karlsen, Karin Lindahl, Elisabeth Kleppa, Anni Assing Winckelmann, Pascal Brugger-Synnes, Hans Erling Simonsen, Jan Svendsen, Niklas K. Björkström, Dag Henrik Reikvam, Asgeir Johannessen

**Affiliations:** 1 Department of Infectious Diseases, Vestfold Hospital, Tønsberg, Norway; 2 Institute of Clinical Medicine, Faculty of Medicine, University of Oslo, Oslo, Norway; 3 Center for Infectious Medicine, Department of Medicine Huddinge, Karolinska Institutet, Karolinska University Hospital, Stockholm, Sweden; 4 Department of Infectious Diseases, Akershus University Hospital, Lørenskog, Norway; 5 Aklilu Lemma Health Research Institute, Addis Ababa University, Addis Ababa, Ethiopia; 6 Department of Infectious Diseases, Oslo University Hospital, Oslo, Norway; 7 Medical Department, St. Paul’s Hospital Millennium Medical College, Addis Ababa, Ethiopia; 8 University Health Network, University of Toronto, Toronto, Canada; 9 Department of Infectious Diseases, Copenhagen University Hospital – Hvidovre, Hvidovre, Denmark; 10 Department of Clinical Medicine, Faculty of Health and Medical Sciences, University of Copenhagen, Copenhagen, Denmark; 11 Medical Department, Vestre Viken Hospital, Drammen, Norway; 12 Department of Clinical Sciences, University of Bergen, Bergen, Norway; 13 Department of Gastroenterology, Stavanger University Hospital, Stavanger, Norway; 14 Department of Medicine, Ålesund Hospital, Ålesund, Norway; 15 Department of Infectious Diseases, Nordland Hospital, Bodø, Norway; 16 Vestre Viken Hospital, Bærum, Norway

**Keywords:** Hepatitis B virus, Treatment discontinuation, Entecavir, Tenofovir, Functional cure, Soluble serum proteins

## Abstract

**Background & Aims:**

Hepatic flares frequently occur after nucleos(t)ide analogue (NA) cessation in patients with chronic hepatitis B (CHB) and can be beneficial (‘good flares’) or harmful (‘bad flares’). We characterised flares after NA cessation aiming to identify predictors and immunological correlates of good and bad flares.

**Methods:**

This study was nested in the prospective Nuc-Stop study, in which 127 patients with e-antigen negative CHB discontinued NA treatment with a 36-month follow-up. Flares were defined as an alanine aminotransferase (ALT) increase >2 × the upper limit of normal or >2 × baseline. Predictors of flares were identified by logistic regression. In 32 patients with flares without treatment restart, we compared clinical characteristics and soluble immune marker profiles of good flares (HBsAg loss or >1 log_10_ decline or sustained virological control) and bad flares (neither HBsAg decline nor virological control).

**Results:**

Flares occurred in 58.3% of patients. Age (per 1-year increment; adjusted odds ratio [aOR], 1.07; 95% confidence intervals [CI] 1.02–1.12) and end-of-treatment HBsAg level (per 1 log_10_ IU/ml increment; aOR, 2.09; 95% CI 1.20–3.62) independently predicted flares. Good flares displayed less ALT variability after the initial spike than bad flares (standard deviation 9.7 *vs.* 22.7 U/L, *p* = 0.002). Analysis of soluble immune markers confirmed distinct clusters for good and bad flares at end-of-treatment, with higher serum levels of specific proteins in good flares (e.g. IL-13, TRAIL) and bad flares (*e.g.* CXCL11, OPG, TNF).

**Conclusions:**

Flares occurred in over half the patients after NA cessation and were associated with age and end-of-treatment HBsAg levels. ALT variability following the initial flare and soluble marker profiles might serve as prognostic factors and distinguish good from bad flares.

**Impact and implications:**

NA cessation in HBeAg-negative CHB may facilitate functional cure, but hepatic flares are common and potentially detrimental, making careful patient selection essential. In this prospective, multicentre trial, flares occurred in over half of the patients, with severe flares in 12.6%. Increasing age and higher end-of-treatment HBsAg levels were predictors of flares and should be considered when deciding on treatment discontinuation. Close monitoring, particularly during the first 6 months, is critical for safe patient management, whereas ALT variability following the initial flare and soluble immune markers may help differentiate good from bad flares and guide retreatment decisions.

**Clinical Trials Registration (for the Nuc-Stop study):**

NCT03681132.

## Highlights

• Hepatic flares are common after nucleos(t)ide analogue (NA) cessation.
• Flares predominantly occur within the first 6 months after NA cessation.
• Older age and higher HBsAg levels predict flares.
• ALT variability may help distinguish good flares from bad flares.
• Soluble marker profiles differ in good *vs.* bad flares.

## Graphical abstract

**Figure:**
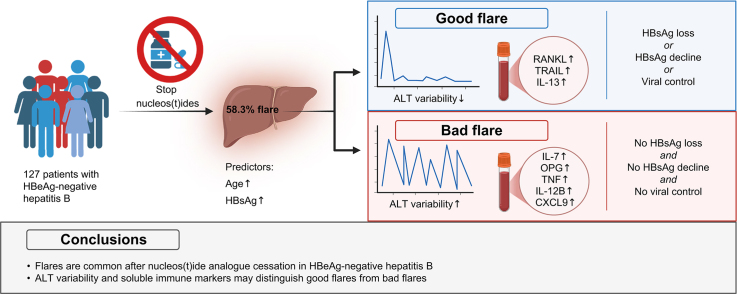

## Introduction

Chronic hepatitis B (CHB) infection affects ∼254 million people worldwide, causing an estimated 1.3 million deaths annually, primarily as a result of cirrhosis and hepatocellular carcinoma (HCC).^1^ Treatment with nucleos(t)ide analogues (NAs) suppresses viral replication and halts disease progression but rarely leads to functional cure defined as hepatitis B surface antigen (HBsAg) loss.^2^^,^^3^ Finite NA treatment is gaining interest, with several studies demonstrating that functional cure can be achieved following NA cessation in patients with HBeAg-negative CHB.[2], [3], [4], [5], [6] Functional cure reduces the risk of fibrosis progression and HCC beyond that achieved by ongoing NA therapy with full HBV DNA suppression.^7^

Treatment withdrawal, however, is not without risks and hepatic flares are common following NA cessation. These flares represent exacerbations of liver inflammation, typically recognised by a sudden rise in alanine aminotransferase (ALT) levels, and can be detrimental, potentially leading to liver injury, hepatic decompensation, or even death.[8], [9], [10] However, it has been proposed that flares may be part of a beneficial immune response with an association between flares and the transition to an inactive carrier state or functional cure.^2^^,^^5^^,^^11^ Contrary to this latter hypothesis, recent studies have indicated that flares have no impact on functional cure, and that functional cure may even be more prevalent among patients who do not experience a flare.^12^^,^^13^

Soluble immune markers (SIMs) have previously been reported to be associated with virological relapse and HBsAg loss in patients with chronic HBV infection undergoing treatment cessation.[14], [15], [16] Yet, definitions of SIM profiles associated with distinct flares that lead to either HBV control or persistence are lacking.

Thus, the existing knowledge about flares and their underlying immunological mechanisms and clinical consequences, is still limited. A better understanding of this critical phase of CHB infection may enhance our understanding of cure and liver disease progression, and enable clinicians to predict which patients may benefit from treatment discontinuation and when to restart, thereby facilitating more tailored treatment strategies.^11^^,^^17^

In this study, we aimed to characterise hepatic flares and identify clinical, biochemical, virological, and immunological predictors and correlates of good *vs*. bad flares after NA cessation in a prospective trial of patients with HBeAg-negative CHB.

## Materials and methods

### Study design and participants

This study was nested in the Nuc-Stop study, a randomised, prospective, multicentre trial originally designed to investigate how two different strategies for retreatment (high-threshold and low-threshold) after NA cessation affected the likelihood of achieving functional cure.^18^

As previously described, the Nuc-Stop study enrolled 127 patients with CHB who were HBeAg-negative, without cirrhosis, and who had been continuously virally suppressed on NA therapy for at least 24 months.^18^ All participants were HBeAg-negative at start of antiviral therapy. Participants were recruited between 2018 and 2020 from 11 centres in Norway, Sweden, Denmark, and Ethiopia. After stopping NA treatment, they were followed for 36 months with pre-defined criteria for restarting NA according to randomisation group:^18^

• high-threshold: ALT >100 U/L for >4 months without decline, or ALT >400 U/L for >2 months;
• low-threshold: HBV DNA >2,000 IU/ml and ALT >80 U/L.

NA treatment was restarted in both groups if:

• ALT > 800 U/L;
• bilirubin >38 mmol/L or international normalised ratio (INR) ≥1.4, in two consecutive samples, and ALT >80 U/L at confirmation;
• cirrhosis diagnosed by elastography.

Study visits were performed at end-of-treatment (EOT), after 4, 8, and 12 weeks and every 3 months thereafter. In the event of a flare, closer follow-up was initiated with controls every 1–2 weeks. Each study visit included clinical evaluation and blood tests.

### Definitions

Hepatic flares were defined as an ALT increase >2 × the upper limit of normal (ULN) or 2 × the baseline value and classified into three severity categories:

• mild flares: ALT 2–5 × ULN or 2–5 × baseline;
• moderate flares: ALT 5–20 × ULN or 5–20 × baseline;
• severe flares: ALT >20 × ULN or >20 × baseline.

Time to a flare was defined as the duration (in months) from EOT to the first occurrence of a biochemical flare, defined as ALT above 2 × ULN/baseline. This definition was applied consistently to enable comparison of the time to mild, moderate, and severe flares.

HBsAg loss was defined as quantitative HBsAg (qHBsAg) below the detection limit of the assay (<0.05 IU/ml). The composite endpoint HBsAg loss/decline was defined as qHBsAg below the detection limit or >1 log_10_ decline 36 months after NA cessation. No HBsAg decline was defined as <0.5 log_10_ decline in qHBsAg after 36 months.

Sustained off-therapy virological control was defined as off-therapy HBV DNA <2,000 IU/ml (with any ALT value) at all study visits during year three after NA cessation.

Flares were then classified into good flares or bad flares:

• good flares: flares occurring in patients who subsequently achieved HBsAg loss/decline or sustained off-therapy virological control;
• bad flares: flares occurring in patients with no subsequent HBsAg loss/decline and absence of off-therapy virological control.

### Laboratory analyses

Laboratory analyses were performed as previously described.^18^ Routine analyses, including ALT measurements, were conducted locally at the participating study centres, whereas qHBsAg was processed in a single batch at Oslo University Hospital, Oslo, Norway, with the Elecsys HBsAg II Quant assay (Roche Diagnostics GmbH, Mannheim, Germany) on the Roche Cobas e801 platform, according to the manufacturer’s instructions. An ALT level of 40 U/L was considered the ULN across all sites. Serum collected for biobanking was stored at -80 °C.^18^ Serum samples from EOT, 3, 6, and 24 months were thawed, plated on non-skirted 96-well plates (ThermoFisher Scientific, Waltham, MA, USA), and quantified for the presence of 92 soluble proteins using the targeted OLINK Inflammation panel (OLINK AB, Uppsala, Sweden).

### Statistical analyses

Continuous variables were presented as medians with IQRs, whereas categorical variables were presented as counts and percentages. The distribution of peak ALT across groups was analysed using the Kruskal–Wallis test, with *post hoc* pairwise comparisons by Dunn’s test and Bonferroni correction to account for multiple comparisons. The Mann-Whitney *U* test was used to compare the time to first flare between patients treated with tenofovir and entecavir.

The cumulative incidence of flares was evaluated using Kaplan-Meier survival analysis, and differences between groups were assessed using the log-rank test. Logistic regression analyses were performed to identify predictors of hepatic flares, with unadjusted and adjusted odds ratios (ORs and aORs) calculated and presented alongside 95% confidence intervals (CIs) and *p* values. Fisher’s exact test was applied to analyse the association between flare severity and HBsAg loss/decline, and the association between EOT HBsAg level and HBsAg loss/decline.

Comparisons of good and bad flares were performed on the subset of patients who experienced a flare without restarting treatment. Based on the safety criteria of the trial, all patients with an ALT increase to >800 U/L restarted treatment immediately and were therefore excluded from this sub-analysis. Levene’s test was used to assess the ALT variability and HBV DNA variability following the initial flare, and ALT and HBV DNA values recorded at or before the first flare, as well as ALT and HBV DNA values after HBsAg loss, were excluded from the analysis. Mann-Whitney *U* test was used to compare peak ALT and EOT HBsAg level between good and bad flares. Fisher’s exact test was used to compare the proportions who discontinued tenofovir and entecavir between patients with good and bad flares.

Statistical analyses (except OLINK data) were performed using Stata version 16.1 (StataCorp, College Station, TX, USA). OLINK data were analysed using the packages OLINKAnalyze (v4.2.0), dplyr (v1.1.4), tidyr (v1.3.1), tibble (v3.2.1), pheatmap (v1.0.12), RColorBrewer (v1.1.3), mixOmics (v6.30.0), and ggplot2 (v3.5.1) in R (v4.4.2, R Foundation for Statistical Computing, Vienna, Austria) to generate heatmap and sparse partial least squares discriminant analysis (sPLS-DA) plots. Normalised expression values (NPX) for specific proteins were visualised in GraphPad Prism (v.10, GraphPad Software, San Diego, CA, USA) and statistical differences were assessed using non-parametric Mann-Whitney *U* test or paired Wilcoxon test for protein expression.

### Ethical considerations

This study was performed in accordance with the Declaration of Helsinki and the International Conference on Harmonization of Good Clinical Practice. The protocol was approved by the National Ethics Committees and Medical Agencies of all participating countries, and by the Data Protection Officials at each study site. All participants provided written informed consent. The Nuc-Stop study is registered in ClinicalTrials.gov (NCT03681132).

## Results

### Study population

The cohort included individuals of African, Asian, and European descent, and all HBV genotypes were represented (Table 1). Tenofovir was the most commonly used NA (76.4%), primarily as tenofovir disoproxil fumarate. The median duration of NA therapy before cessation was 45 months (IQR 32–76), and the median EOT HBsAg level was 2,213 IU/ml (IQR 762–6,105). In total, 37 patients (29.1%) restarted treatment within 36 months, including 35 (47.3%) among those who experienced a flare. The two patients who restarted treatment without experiencing a flare did so because of pregnancy and relocation abroad.

**Table 1:** Demographics and baseline characteristics (N = 127).

	No flare (n = 53)	Flare (n = 74)
Age (years)	42 (35–47)	45 (39–54)
Men	36 (67.9)	50 (67.6)
Ethnicity		
African	26 (49.1)	26 (35.1)
Asian	20 (37.7)	35 (47.3)
European	7 (13.2)	13 (17.6)
Genotype		
A	17 (32.1)	12 (16.2)
B	4 (7.6)	13 (17.6)
C	5 (9.4)	13 (17.6)
D	17 (32.1)	24 (32.4)
E	2 (3.8)	8 (10.8)
Unknown	8 (15.1)	4 (5.4)
BMI (kg/m^2^)	25.2 (22.2–27.0)	23.9 (21.6–26.6)
Antiviral medication		
Tenofovir∗	39 (73.6)	58 (78.4)
Entecavir	14 (26.4)	16 (21.6)
Time on NA (months)	44 (32–62)	52 (33–78)
ALT	33 (25–44)	28 (21–39)
qHBsAg (IU/ml)	1,294 (576–4,872)	2,909 (796–7,620)
qHBsAg level (IU/ml)		
≤100	8 (15.1)	4 (5.4)
100–1,000	12 (22.6)	17 (23.0)
>1,000	33 (62.3)	53 (71.6)
Liver fibrosis stage before NA therapy		
F0/F1	32 (60.4)	39 (52.7)
F2	9 (17.0)	11 (14.9)
F3	5 (9.4)	10 (13.5)
Missing	7 (13.2)	14 (18.9)

Data are presented as median (IQR) or n (%).

ALT, alanine aminotransferase; N, number of patients; NA, nucleos(t)ide analogue; qHBsAg, quantitative hepatitis B surface antigen.

∗ Tenofovir disoproxil fumarate (n = 90), tenofovir alafenamide fumarate (n = 6), and tenofovir disoproxil fumarate/emtricitabine (n = 1).

### Frequency, timing, and severity of flares

Flares were observed in 74 (58.3%) patients of whom 38 (29.9%) had mild flares, 20 (15.5%) moderate flares, and 16 (12.6%) severe flares (Fig. S1). The median peak ALT among patients with a flare was 139 (IQR 89–340) U/L. Most flares occurred early; 59.5% occurred within the first 3 months after EOT. Early flares were more severe than later flares based on peak ALT (*p* = 0.034), driven by higher ALT in flares within 3 months (*p* = 0.018) and 3–6 months (*p* = 0.026) compared with those after 12 months (Fig. S2).

**Fig. 1:**
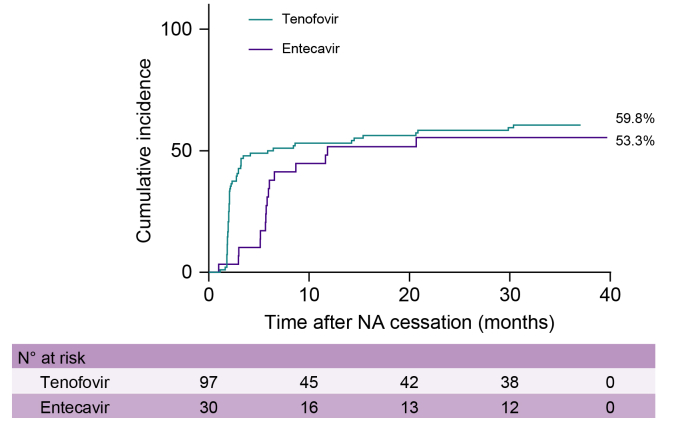
Cumulative incidence of flares after NA cessation by treatment type. Cumulative incidence of flares in patients who discontinued tenofovir *vs.* entecavir. No significant difference was observed between the groups (log-rank test, *p* = 0.230). Patients were censored at retreatment or withdrawal. NA, nucleos(t)ide analogue.

The median time from EOT to first flare was significantly shorter for patients who discontinued tenofovir (2.1 [IQR 1.9–3.2] months) than entecavir (5.8 [IQR 5.1–7.6] months) (*p* = 0.001) (Fig. 1). Over 36 months, however, there was no significant difference in the cumulative incidence of flares between patients who had been treated with tenofovir (59.8%) and entecavir (53.3%) (*p* = 0.23).

All severe flares occurred in the tenofovir group. The median time from EOT to first flare was 2.0 (IQR 1.8–3.0) months for patients who experienced a severe flare, and all had their first flare within 6 months. The median peak ALT among patients with a severe flare was 936 (IQR 617–1,705) U/L. After restart of treatment, ALT declined to normal levels and HBV DNA became fully suppressed in all patients. None of the patients developed liver decompensation.

### Predictors of flares

In univariable analysis, age, EOT HBsAg level, and HBV genotype were associated with hepatic flares (Table 2). In multivariable analysis, only age (per 1-year increment; aOR, 1.07; 95% CI 1.02–1.12) and EOT HBsAg level (per 1 log_10_ IU/ml increment; aOR, 2.09; 95% CI, 1.20–3.62) remained independent predictors of flares. There was no significant association between flares and sex, ethnicity, BMI, type of antiviral treatment, or treatment duration.

**Table 2:** Predictors of hepatic flares.

Variable	Unadjusted			Adjusted		
	OR	95% CI	*p* value	OR	95% CI	*p* value
Age (years)	1.05	1.01–1.09	0.02	1.07	1.02–1.12	0.01
Sex (male *vs*. female)	0.98	0.46–2.09	0.97			
Ethnicity (Asian *vs*. non-Asian)	1.40	0.68–2.88	0.36			
Genotype (B/C *vs*. other)	2.36	0.98–5.68	0.05	2.29	0.87–6.02	0.09
BMI (kg/m^2^)	0.97	0.89–1.06	0.47			
Tenofovir *vs.* entecavir	0.77	0.34–1.75	0.53			
Treatment duration (months)	1.00	0.99–1.01	0.70			
qHBsAg (log_10_ IU/ml)	1.61	1.07–2.42	0.02	2.09	1.20–3.62	0.01

Logistic regression analyses were performed to identify predictors of hepatic flares. Variables with *p* <0.10 in univariate analyses were included in the multivariate model. OR, odds ratio; qHBsAg, quantitative hepatitis B surface antigen.

### Patient outcome after flares

Among the 127 study participants, 11 (8.7%) achieved HBsAg loss and 6 (4.7%) remained HBsAg positive but had a >1 log_10_ IU/ml HBsAg decline within 36 months. HBsAg loss/decline occurred in nine of 53 patients without flares (17.0%), five of 58 with mild/moderate flares (8.6%), and three of 16 with severe flares (18.8%) (Fig. S3). There was no significant association between flare severity and HBsAg loss/decline (*p* = 0.356).

Table 3 shows flares and HBsAg loss/decline as a function of EOT HBsAg level. HBsAg loss/decline was most common, and flares least common, among patients with EOT HBsAg below 100 IU/ml, and there was a strong association between EOT HBsAg level and HBsAg loss/decline (*p* <0.001).

**Table 3:** EOT qHBsAg in relation to flares and HBsAg loss/decline.

EOT qHBsAg (IU/ml)	Total number of patients n	Flare ALT >2 × ULN/BA n (%)	Flare ALT >5 × ULN/BA n (%)	Flare ALT >20 × ULN/BA n (%)	HBsAg loss/decline∗ n (%)
≤100	12	4 (33.3)	2 (16.7)	1 (8.3)	11 (91.7)
100–1,000	29	17 (58.6)	13 (44.8)	6 (20.7)	4 (13.8)
>1,000	86	53 (61.6)	21 (24.4)	9 (10.5)	2 (2.3)

ALT, alanine aminotransferase; BA, baseline; EOT, end-of-treatment; qHBsAg, quantitative hepatitis B surface antigen; ULN, upper limit of normal.

∗ HBsAg loss/decline was defined as qHBsAg below the detection limit or >1 log_10_ decline 36 months after EOT.

### Good *vs*. bad flares

Thirty-eight patients experienced a flare and did not restart treatment throughout the follow-up period. Of these, 13 were classified as good flares and 19 as bad flares, whereas six did not fit into either category and were excluded from further analyses. Among patients with good flares, three achieved HBsAg loss, three had an HBsAg decline >1 log_10_, and seven had sustained off-therapy virological control. There was no significant difference in NA therapy between those with good and bad flares (proportion tenofovir, 84.6% *vs.* 84.2%, *p* = 1.00); however, the EOT HBsAg level was significantly lower in patients with good flares (median EOT HBsAg 762 *vs.* 4,397, *p* = 0.025).

**Fig. 2:**
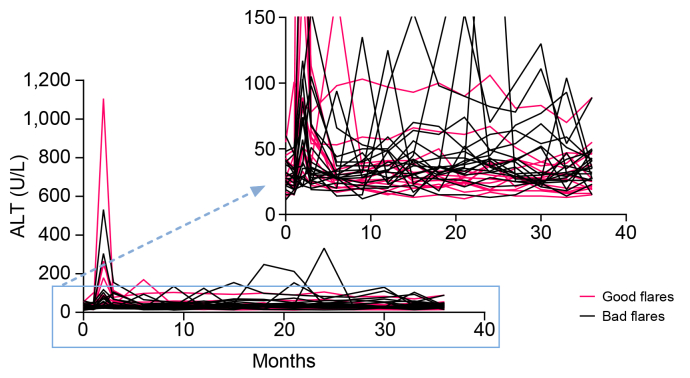
Longitudinal ALT levels in patients with good and bad flares after NA cessation. ALT levels from EOT to 36 months of follow-up are shown for individual patients who experienced flares without restarting treatment classified as good (HBsAg loss/decline or sustained virological control) or bad flares (neither HBsAg loss/decline nor virological control). Good flares are pink (n = 13) and bad flares are black (n = 19). Each line represents one patient. The inset magnifies ALT fluctuations below 150 U/L for greater detail. ALT, alanine aminotransferase; EOT, end-of-treatment; NA, nucleos(t)ide analogue.

Following the initial flare, patients with good flares displayed less ALT fluctuations compared with those with bad flares (Fig. 2). The mean SD of ALT was 9.7 ± 5.8 U/L in patients with good flares *vs.* 22.7 ± 24.3 U/L in patients with bad flares (*p* = 0.002). The median peak ALT, however, was similar between the two groups (89 U/L; IQR 52–179 *vs.* 103 U/L; IQR 74–154; *p* = 0.66). Variability in HBV DNA was not significantly different between the groups (Fig. S4); the mean SD was 0.5 ± 0.2 log_10_ IU/ml in patients with good flares *vs.* 0.9 ± 0.9 log_10_ IU/ml in patients with bad flares (*p* = 0.066).

### Soluble marker profiles of flares

**Fig. 3:**
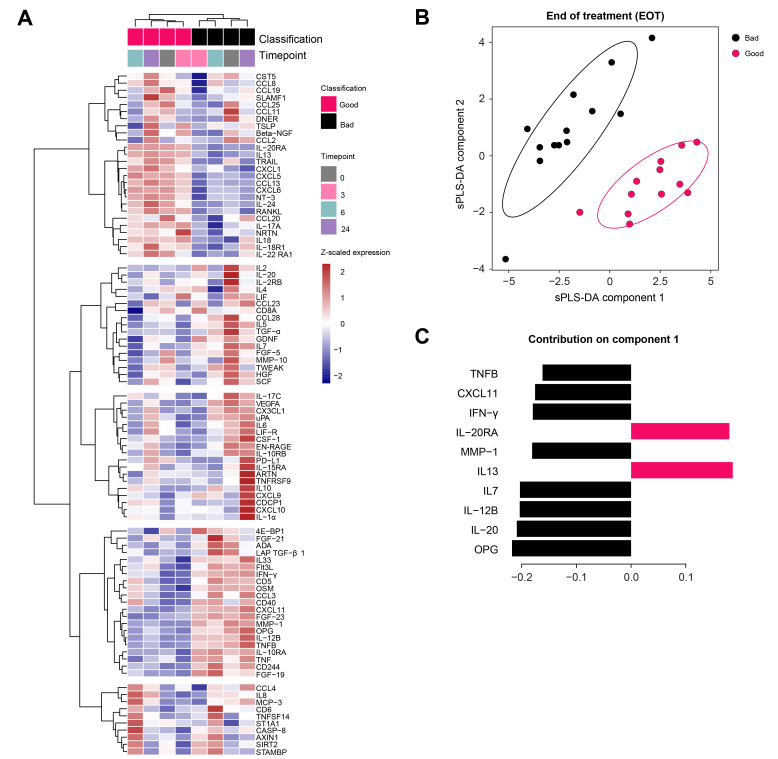
SIMs distinguish patients with good and bad flares. (A) Heatmap of SIM expression in good (pink) and bad (black) flares at EOT (grey), 3 months (pale pink), 6 months (turquoise), and 24 months (purple). Row-scaled SIM expression; hierarchical clustering (Ward’s method). (B) sPLS-DA at EOT based on normalised protein expression. Each point represents one patient, good flares (pink, n = 12), bad (black, n = 13). Ellipses indicate the 75% confidence region. (C) Top 10 features contributing to component 1 of good (pink) and bad (black) flares, ranked by discriminative power. EOT, end-of-treatment; SIMs, soluble immune proteins; sPLS-DA, sparse partial least squares discriminant analysis.

To further explore how good and bad flares differed in terms of their SIMs, 92 defined soluble proteins, commonly upregulated during inflammation, were analysed at EOT and at 3, 6, and 24 months using OLINK technology. In total, SIMs were determined in 25 patients with flares, of whom 12 were classified as good and 13 as bad. The classification into good and bad flares was the main distinction for observed soluble signatures with certain proteins being more abundant in the serum of patients with good flares (*e.g.* IL-13, receptor activator of nuclear factor kappa-B ligand [RANKL] and tumour necrosis factor-related apoptosis-inducing ligand [TRAIL]), whereas others were more prominent in bad flare patients (*e.g.* C-X-C motif chemokine ligand [CXCL])11, IL-12B and osteoprotegerin [OPG]), irrespective of the measured timepoint (Fig. 3A). Other soluble molecules displayed distinct levels at certain timepoints, for example CXCL9 and CXCL10 which started to increase after 3 months and peaked at 24 months within bad flare categorised patients. Two distinct clusters for good and bad flares were identified using sPLS-DA clustering analysis at EOT (Fig. 3B), suggesting that SIMs may indicate different outcomes even before treatment cessation. The main contributors to detected differences in good *vs.* bad flare outcomes included OPG, IL-20, IL-12B, IL-7 IL-13, CXCL11, and interferon-gamma (IFN-γ) (Fig. 3C).

**Fig. 4:**
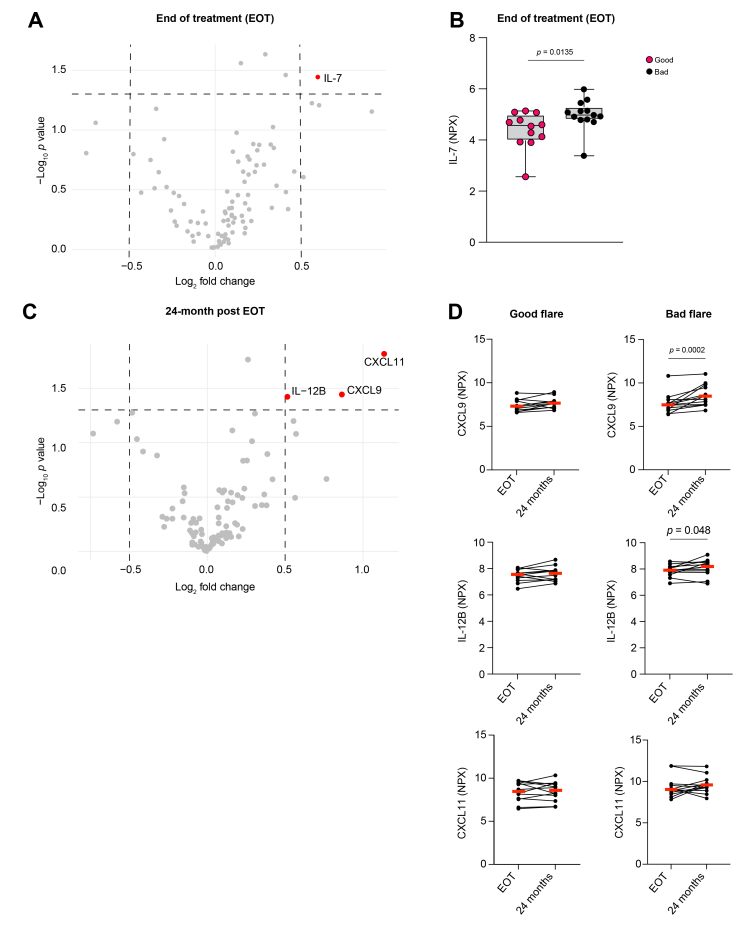
Differential expression of SIMs early and late after EOT. (A) Volcano plot showing differential SIM expression between good and bad flares at EOT. Each point represents one protein (n = 92). Dashed lines indicate the thresholds for significance with fold change (+/- 0.5) and -log_10_*p* value (*p* = 0.05). Significant proteins are red, non-significant grey. (B) NPX for IL-7 at EOT for good (pink, n = 12) and bad (black, n = 13) flares. Boxes represent the IQR; horizontal lines medians. ∗*p* = 0.0135 (Mann-Whitney *U* test). (C) Volcano plot showing differential SIM expression between good and bad flares at 24 months after EOT (same thresholds as in part A). (D) NPX values for CXCL9 (upper panel), IL-12B (middle panel), and CXCL11 (lower panel) at EOT and 24 months after EOT for good (left; n = 12) and bad (right; n = 13) flares. Lines connect NPX values of the same patient. Red lines indicate the median. ∗∗∗*p* = 0.002, ∗*p* = 0.048 (Wilcoxon matched signed-rank test). CXCL, C-X-C motif chemokine ligand; EOT, end-of-treatment; NPX, normalised expression values; SIMs, soluble immune proteins.

Given the distinct SIM profiles between patients classified as having good *vs.* bad flares, proteins that differed significantly at EOT between these two clinical groups were subsequently determined (Fig. 4A). IL-7 was the only soluble marker significantly associated with bad flares at EOT with higher levels of soluble IL-7 in the sera of bad flare patients (Fig. 4A and B). At later timepoints there were no significant differences in soluble IL-7 levels between the groups (data not shown), indicating soluble IL-7 as a potential early predictive marker for the development of hepatic flares without subsequent HBsAg loss. The SIM profile changed at later stages after EOT, with CXCL9, CXCL11, and IL-12B being significantly upregulated in serum of bad flare patients at 24 months (Fig. 4C). When assessing the dynamic changes of those markers from EOT to 24 months, CXCL9 and IL-12B showed a significant increase from EOT to 24 months in bad but not good flare patients, whereas changes of CXCL11 levels were non-significant (Fig. 4D).

## Discussion

In this prospective trial, more than half of the patients with HBeAg-negative CHB experienced a flare after cessation of NA therapy. Increasing age and higher EOT HBsAg levels were independent predictors of flares. Importantly, patients with good flares displayed less ALT variability following the initial flare than patients with bad flares, and the two groups exhibited distinct SIM profiles, suggesting different underlying immunological mechanisms.

Following the initial flare, patients with good flares displayed less fluctuation in ALT levels compared with those with bad flares. To our knowledge, ALT variability as a prognostic factor following NA cessation has not been previously investigated. Despite the small sample size (13 patients with good flares and 19 with bad flares), we observed a statistically significant difference in ALT variability between the two groups, although the peak ALT was similar. Natural history studies have shown that high ALT variability in treatment-naïve HBeAg-negative CHB is a marker of hepatic necroinflammation, potentially leading to progressive fibrosis, and it is likely that the ALT variability observed in our study reflects the same underlying disease process.^19^ Our findings should be explored in larger prospective studies, and if confirmed, ALT variability could serve as a prognostic factor and guide decisions on treatment reinitiation.

A flare incidence of 58.3% (28.4% exceeding 5 × ULN/baseline) in our study is in line with the retrospective RETRACT-B study, which reported a 5-year cumulative incidence of hepatic flares (defined as ALT ≥5 × ULN) of 33%.^13^ Other NA cessation studies have reported hepatic flare rates ranging from 16% to 52% over follow-up periods of 18-24 months.^6^^,^^12^^,^^20^^,^^21^ Comparing flare incidence across studies is challenging as a result of differences in flare definitions, follow-up procedures, and retreatment criteria. However, we believe that our study, with its prospective design, close follow-up, and inclusion of diverse ethnicities and genotypes, provides a more reliable estimate of flare incidence than many previous retrospective or observational studies.

Increasing age and higher EOT HBsAg levels were independent predictors of flares, consistent with findings from the retrospective RETRACT-B study^13^ and an observational study by Liu and colleagues.^20^ However, unlike these studies, we did not identify male sex or tenofovir treatment as independent predictors of flare. It is well established that entecavir-associated flares tend to occur later than tenofovir-associated flares.^20^^,^^22^ A possible explanation for not identifying tenofovir as a predictive factor in our study, is that our closer follow-up schedule enabled better detection of later-onset flares in entecavir treated patients, which may have gone undetected in the RETRACT-B study because of longer follow-up intervals of up to 6 months. Similarly, the shorter observation period in the study by Liu and colleagues^20^ (12–24 months) may have led to underdetection of late-onset flares in patients who stopped entecavir.

We identified distinct soluble immune signatures in the serum of patients experiencing good and bad flares. High levels of the decoy receptor OPG were one of the contributing SIMs distinguishing good and bad flares at EOT. High OPG has previously been associated with more advanced liver damage and cirrhosis, while higher levels of its ligand, the chemokine RANKL, as well as a low ratio of OPG/RANKL levels, were more pronounced in earlier stages of liver disease.^23^^,^^24^ Indeed, RANKL was one of the main components distinguishing good and bad flares at 3 months after EOT, indicating that the OPG/RANKL ratio could be of use for further HBV treatment cessation trails to predict patient outcome.

Soluble IL-7 was the only SIM that was significantly different at EOT, with higher levels in patients subsequently developing hepatic flares without HBsAg loss/decline. Low IL-7 levels have previously been associated with lower incidences of viral relapse following NA treatment cessation in CHB.^16^ One major difference between the herein presented study and the previous publication is the definition of patient groups. Wübbolding *et al.*^16^ defined the patients as viral relapse or no relapse based on the HBV DNA levels 24 weeks after EOT, whereas our definition is based on a 36-month composite outcome of HBsAg loss/decline and virological control. Further prospective studies should explore the potential use of EOT IL-7 as a prognostic marker in individuals considered for NA withdrawal.

Chemokines, including CXCL9, 10, and 11, have previously been shown to be associated with hepatic flares,[25], [26], [27] and in our study their levels at 24 months after EOT were higher in patients with bad flares compared to patients with good flares. Chemokines recruit immune cells to the liver, including immune cells that do not specifically recognise HBV antigens and hence contribute to liver damage and hepatic flares.^28^^,^^29^ Although CXCL9, 10, and 11 levels where low in both clinical groups at EOT, levels of all three chemokines increased more profoundly in the bad flare patient group. Importantly, soluble protein levels of CXCL9, 10, and 11 remained high at 24 months after EOT in the bad flare patient group, indicating ongoing immune cell recruitment to the liver, potentially contributing to liver damage while failing to effectively clear HBV-infected hepatocytes.

Similar to other studies, we observed an elevation in tumour necrosis factor (TNF) levels after EOT, which was more pronounced in patients experiencing hepatic flares without HBsAg loss/decline.^30^ The secretion of TNF by HBV-specific CD4^+^ T cells has been associated with higher disease severity in chronic HBV infection compared with the sole secretion of IFN-γ which was associated with a higher likelihood of clearing HBV infection.^31^ Additionally, TNF levels have been described to be increased in respect to corresponding ALT levels in a liver injury rat model.^32^ Hence, ALT variability as well as TNF serum levels could provide guidance on whether patients with HBV infection undergoing treatment cessation should restart NA treatment to prevent further liver damage.

In the present study, there was no evidence indicating that flares *per se* improved the likelihood of HBsAg loss/decline. This finding contradicts the ‘beneficial flare’ hypothesis, which proposes that flares reflect a favourable immune response, and may promote transition to inactive disease or functional cure. Our findings are consistent with recent studies by Feld *et al.*^12^ and Dongelmans *et al.*,^13^ which found no significant association between flares and subsequent HBsAg loss. The concept of good flares and bad flares remains an area of debate, with varying definitions proposed.^19^^,^^33^ In our study, we proposed an outcome-based definition to distinguish between good and bad flares, as we believe this differentiation is essential both for the individual patient but also for research aiming to inform future treatment strategies.

Our study had some limitations. First, we cannot exclude the possibility that flares may have occurred between scheduled follow-up visits and gone undetected. Second, HBsAg loss was rare, and a larger sample size and/or a longer follow-up period would have increased statistical power and robustness of our conclusions. To mitigate this, we used a combined endpoint of HBsAg loss and >1 log_10_ HBsAg decline. Third, comparisons between good and bad flares were restricted to patients who did not resume treatment, potentially introducing bias by excluding those with the most severe flares. This may—if anything—have led to an underestimation of the true differences between good and bad flares, and we believe the observed difference in ALT variability is valid, which is supported by distinct soluble marker profiles, suggesting differences in the underlying immunological mechanisms. Finally, patients were enrolled in this study after ≥24 months of continuous viral suppression on NA therapy. Therefore, virological and biochemical parameters before study enrolment were unavailable. This also applies to the exact consolidation period, for which the total duration of NA therapy was used as a surrogate.

Despite these limitations, our study had several strengths. First, the inclusion of diverse ethnicities and HBV genotypes, including African patients who have been underrepresented in previous NA stop studies, enhances the generalisability of our findings. Second, the rigorous prospective design enabled us to capture more flares than retrospective studies. Third, the intensive follow-up protocol, with visits scheduled every 1–2 weeks once a flare was identified, may have contributed to the absence of liver decompensation, as timely retreatment was initiated. Fourth, our flare definition, which included an absolute ALT threshold of 2x ULN as well as a relative ALT threshold of 2 × baseline, allowed us to identify patients earlier and more accurately, as normal ALT values vary significantly depending on factors such as age, sex, body weight, and ethnicity; hence, liver inflammation may go unnoticed when using a single ALT cut-off.

In conclusion, flares occurred in more than half of the patients with HBeAg-negative CHB who stopped NA therapy in this prospective trial. Increasing age and higher EOT HBsAg levels were identified as independent predictors of flares. Lower ALT variability following the initial flare was associated with good flares, with subsequent HBsAg loss/decline or off-therapy virological control. Finally, good and bad flare patients differed in their SIM profiles with IL-7 distinguishing good and bad flare patients already at EOT. Hence, ALT variability and SIM profiles may be important prognostic factors to inform clinicians about whether to restart NA therapy or not.

## Abbreviations

ALT, alanine aminotransferase; aOR, adjusted odds ratio; BA, baseline; CHB, chronic hepatitis B; CXCL, C-X-C motif chemokine ligand; EOT, end-of-treatment; HCC, hepatocellular carcinoma; IFN-γ, interferon-gamma; INR, international normalised ratio; NA, nucleos(t)ide analogue; NPX, normalised protein expression; OPG, osteoprotegerin; OR, odds ratio; qHBsAg, quantitative hepatitis B e antigen; RANKL, receptor activator of nuclear factor kappa-B ligand; SIM, soluble immune marker; sPLS-DA, sparse partial least squares discriminant analysis; TNF, tumour necrosis factor; TRAIL, tumour necrosis factor-related apoptosis-inducing ligand; ULN, upper limit of normal.

## Authors’ contributions

Designed the study: AJ, DHR, OD. Supervised the study: AJ, DHR, NKB. Recruited and examined the study participants: AJ, DHR, OD, S.A, NB, HD, NW, TS, LH, ES, LNK, KL, EK, AAW, PBS, HES, JS. Performed the OLINK experiments: AN. Data analysis: AJ, DHR, AN, MH. Drafted the manuscript: AJ, DHR, AN, MH. Contributed to editing and finalising the manuscript: all authors.

## Data availability

The data supporting the findings of this study are available upon reasonable request. The data are not publicly available due to the presence of sensitive information that could compromise participant privacy.

## Financial support

This study was funded by 10.13039/501100006095South-Eastern Norway Regional Health Authority (grant no. 2018092 10.13039/100016174AJ and 2021091 10.13039/100016174AJ), Gilead Sciences Nordic Fellowship Program (DHR), Stiftelsen Clas Groschinskys Minnesfonden (MF2552, AN), 10.13039/501100004359Swedish Research Council (2025-06655, AN), 10.13039/501100005416Research Council of Norway (grant no. 336567, DHR), and 10.13039/501100004047Karolinska Institutet. The funders had no role in study design, data collection and analysis, decision to publish, or preparation of the manuscript.

## Conflicts of interest

SA has received honoraria for lectures/educational events from Gilead, MSD and Biogen, has participated in advisory board for Gilead and Ribocure, and reports grants from Gilead and AbbVie. DHR has received research support from Gilead Sciences. AJ has received a research grant from Roche. All other authors declare no conflicts of interest.

Please refer to the accompanying ICMJE disclosure forms for further details.
