# Supplementary material for: Hepatic flares, their immune signatures, and ALT variability after nucleos(t)ide analogue cessation in HBeAg-negative hepatitis B
Source: JHEP Rep. 2026 Apr 29;8(7):101875. doi: 10.1016/j.jhepr.2026.101875 (PMC13315182; doi:10.1016/j.jhepr.2026.101875)
Supplement: Multimedia component 1 [file mmc1.pdf]

Hepatic flares, their immune signatures, and ALT variability after nucleos(t)ide analogue cessation in HBeAg-negative hepatitis B

Marte Holmberg, Annika Niehrs, Olav Dalgard, Nega Berhe, Hailemichael Desalegn, Soo Aleman, Nina Weis, Tore Stenstad, Lars Heggelund, Ellen Samuelsen, Lars Normann Karlsen, Karin Lindahl, Elisabeth Kleppa, Anni Assing Winckelmann, Pascal Brugger-Synnes, Hans Erling Simonsen, Jan Svendsen, Niklas K Björkström, Dag Henrik Reikvam, Asgeir Johannessen

Table of contents

Fig. S1 2

Fig. S2 3

Fig. S3 4

Fig. S4 5

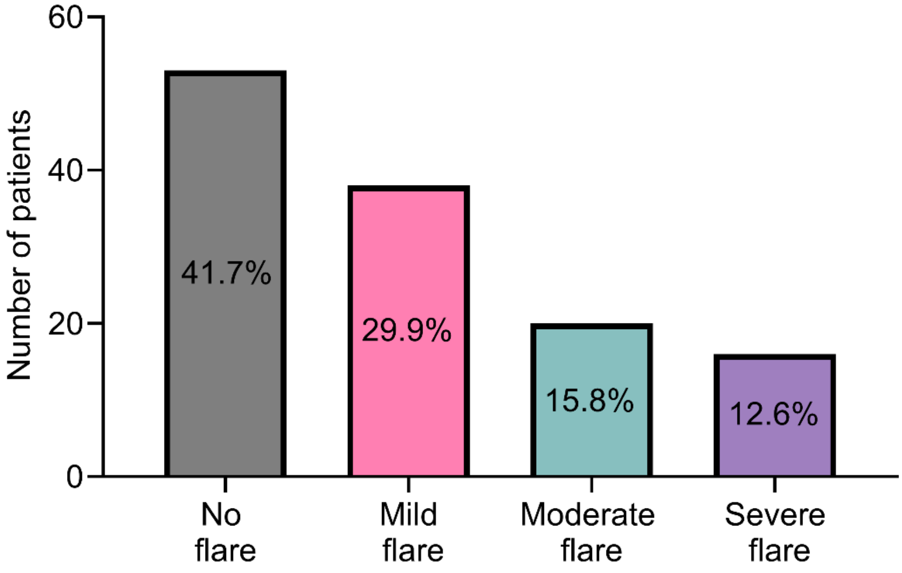

Fig. S1. Distribution of flares after nucleos(t)ide analogue cessation.

In total, 74 (58.3%) of 127 patients experienced a flare.

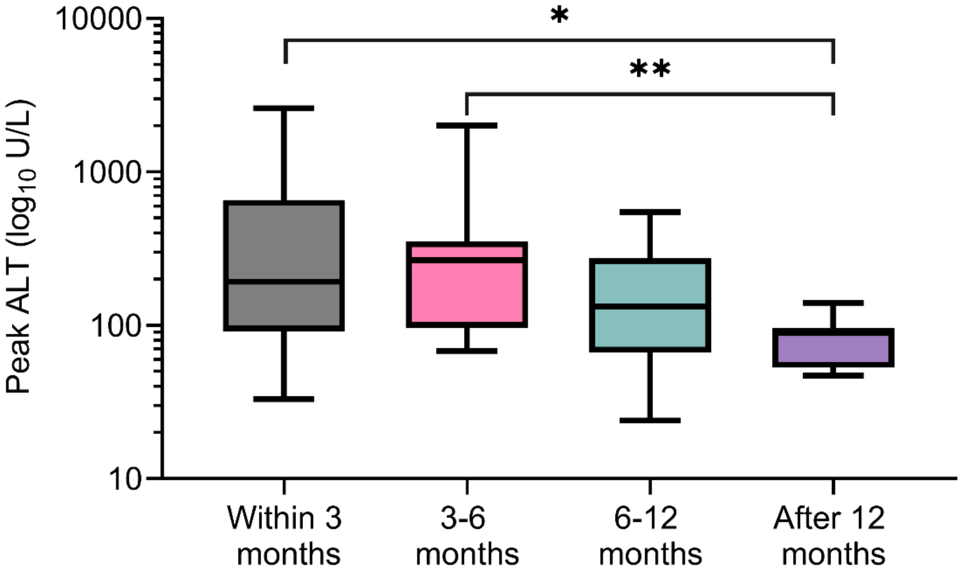

Fig. S2. Severity and timing of flares after nucleos(t)ide analogue cessation in 127 study participants with HBeAg-negative chronic hepatitis B.

Early flares were significantly more severe (i.e. had higher peak ALT) than later flares (Kruskal-Wallis p = 0.034). Dunn's post hoc test showed significantly higher ALT in patients with flares within 3 months (*p = 0.018) and 3–6 months (**p = 0.026) compared to flares occurring after 12 months. No other pairwise comparisons were significant. Boxes represent the IQR; horizontal lines indicate medians. ALT, alanine aminotransferase; IQR, interquartile range.

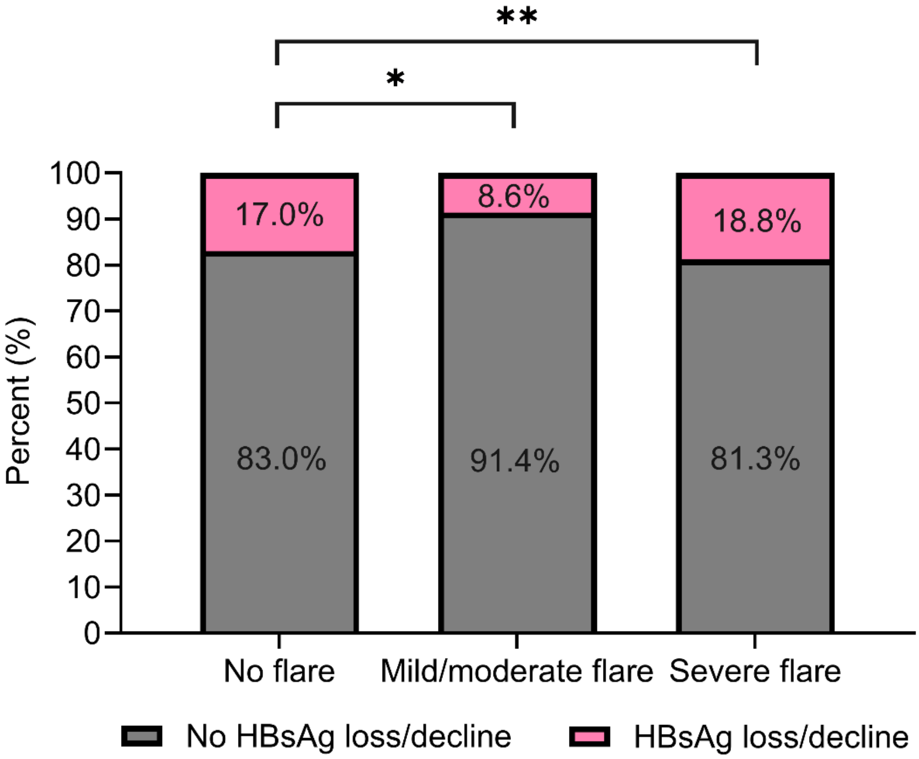

Fig. S3. Flare severity and association with HBsAg loss/decline in 127 study participants with HBeAg-negative chronic hepatitis B.

Proportion of patients with HBsAg loss or >1 log_{10} decline across flare severity groups. For this analysis, mild and moderate flares were combined to increase statistical power. Fisher's exact test showed no significant association between flare severity and HBsAg loss/decline ($^{*}p = 0.254$ and $^{**}p > 0.999$).

A

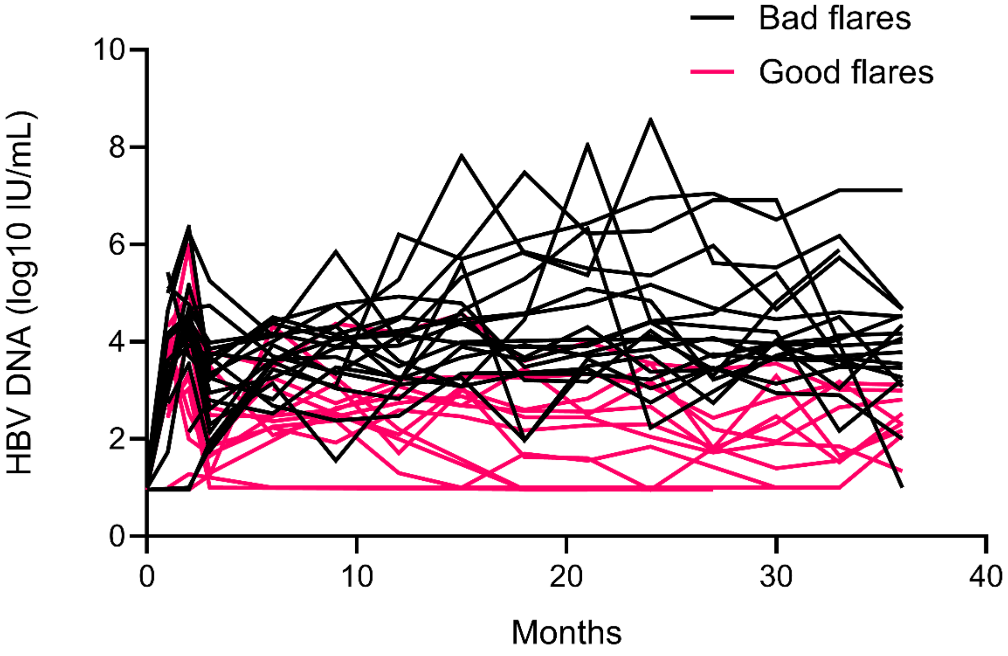

B

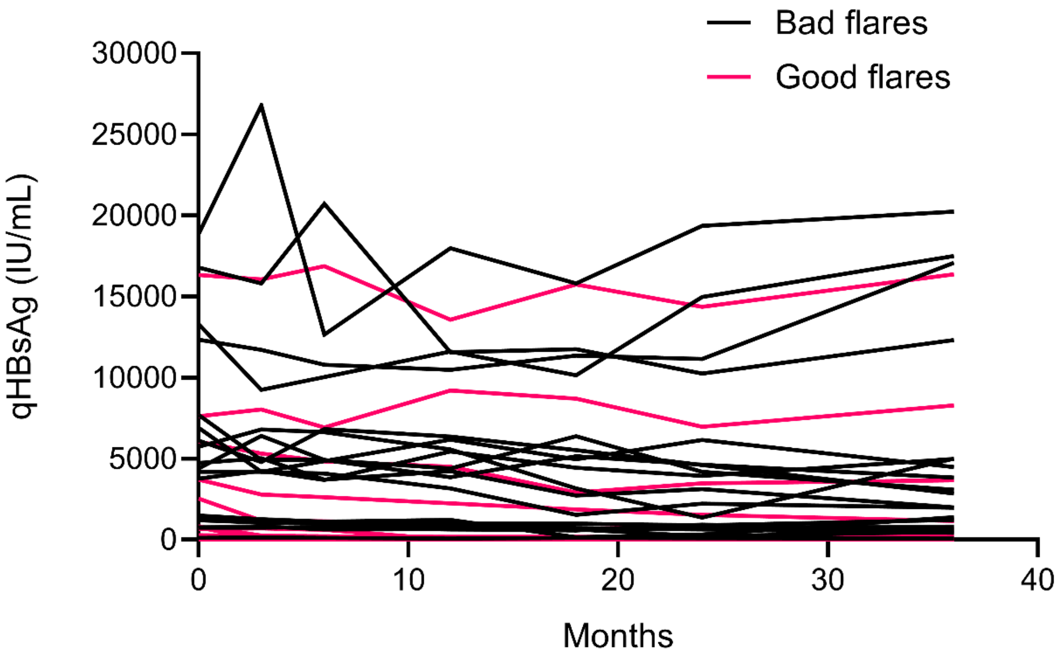

Fig. S4. Longitudinal HBV DNA and qHBsAg in patients with good and bad flares after nucleos(t)ide analogue cessation.

(**A**) HBV DNA (log_{10} IU/mL) from EOT to 36 months of follow-up in individual patients who experienced flares without restarting treatment classified as good (HBsAg loss/decline

or sustained virological control) or bad flares (neither HBsAg loss/decline nor virological control). Good flares are pink (n=13) and bad flares are black (n=19). Each line represents one patient. (**B**) qHBsAg (IU/mL) over the same follow-up period, shown as in panel A. qHbsAg, quantitative hepatitis B surface antigen; EOT, end-of-treatment.
