# Supplementary material for: Hepatic flares, their immune signatures, and ALT variability after nucleos(t)ide analogue cessation in HBeAg-negative hepatitis B
Source: JHEP Rep. 2026 Apr 29;8(7):101875. doi: 10.1016/j.jhepr.2026.101875 (PMC13315182; doi:10.1016/j.jhepr.2026.101875)
Supplement: Multimedia component 2 [file mmc2.docx]

**JHEP Reports**

**CTAT methods**

Tables for a “Complete, Transparent, Accurate and Timely account” (CTAT) are now mandatory for all revised submissions. The aim is to enhance the reproducibility of methods.

- Only include the parts relevant to your study
- Refer to the CTAT in the main text as ‘Supplementary CTAT Table’
- Do not add subheadings
- Add as many rows as needed to include all information
- Only include one item per row

**If the CTAT form is not relevant to your study, please outline the reasons why:**

|  |
| --- |

- 1. **Antibodies**

| **Name** | **Citation** | **Supplier** | **Cat no.** | **Clone no.** |
| --- | --- | --- | --- | --- |
| Not applicable |  |  |  |  |

- 1. **Cell lines**

| **Name** | **Citation** | **Supplier** | **Cat no.** | **Passage no.** | **Authentication test method** |
| --- | --- | --- | --- | --- | --- |
| Not applicable |  |  |  |  |  |

- 1. **Organisms**

| **Name** | **Citation** | **Supplier** | **Strain** | **Sex** | **Age** | **Overall n number** |
| --- | --- | --- | --- | --- | --- | --- |
| Not applicable |  |  |  |  |  |  |

- 1. **Sequence based reagents**

| **Name** | **Sequence** | **Supplier** |
| --- | --- | --- |
| Not applicable |  |  |

- 1. **Biological samples**

| **Description** | **Source** | **Identifier** |
| --- | --- | --- |
| Serum from patients undergoing NA treatment cessation collected at end of treatment, 3, 6, 12, 18, 24 and 36 months post treatment cessation. | Clinical cohort originally described in:  Johannessen A, Reikvam DH, Aleman S, Berhe N, Weis N, Desalegn H, Stenstad T, Heggelund L, Samuelsen E, Karlsen LN, Lindahl K, Pettersen FO, Iversen J, Kleppa E, Bollerup S, Winckelmann AA, Brugger-Synnes P, Simonsen HE, Svendsen J, Kran AB, Holmberg M, Olsen IC, Rueegg CS, Dalgard O. Clinical trial: An open-label, randomised trial of different re-start strategies after treatment withdrawal in HBeAg negative chronic hepatitis B. Aliment Pharmacol Ther. 2024 Aug;60(4):434-445. doi: 10.1111/apt.18147. Epub 2024 Jul 5. PMID: 38970293. |  |

- 1. **Deposited data**

| **Name of repository** | **Identifier** | **Link** |
| --- | --- | --- |
|  |  |  |

- 1. **Software**

| **Software name** | **Manufacturer** | **Version** |
| --- | --- | --- |
| Stata | StataCorp | 16.1 |
| R | R | 4.4.2 |
| GraphPad Prism | GraphPad Software Inc. | 10 |

- 1. **Other (*e.g*. drugs, proteins, vectors etc.)**

|  |  |  |
| --- | --- | --- |
|  |  |  |

- 1. **Please provide the details of the corresponding methods author for the manuscript:**

| Marte Holmberg  Department of Infectious Diseases, Vestfold Hospital, Tønsberg, Norway  Phone: +47 93243267; E-mail: [marte.holmberg@online.no](mailto:marte.holmberg@online.no)  Annika Niehrs  Center for Infectious Medicine, Department of Medicine Huddinge, Karolinska Institutet, Karolinska University Hospital, Stockholm, Sweden  Phone: +46 735018261; Email: [Annika.niehrs@ki.se](mailto:Annika.niehrs@ki.se) |
| --- |

**2.0 Please confirm for randomised controlled trials all versions of the clinical protocol are included in the submission. These will be published online as supplementary information.**

| Not applicable |
| --- |
